# Liquid crystals for organic thin-film transistors

**DOI:** 10.1038/ncomms7828

**Published:** 2015-04-10

**Authors:** Hiroaki Iino, Takayuki Usui, Jun-ichi Hanna

**Affiliations:** 1 Imaging Science and Engineering Laboratory, Tokyo Institute of Technology, J1-2, 4259 Nagatsuta, Midori-ku, Yokohama 226-8503, Japan; 2 Japan Science and Technology Agency (JST), Core Research for Evolutional Science and Technology (CREST), 4-1-8 Hon-cho, Kawaguchi 332-0012, Japan

## Abstract

Crystalline thin films of organic semiconductors are a good candidate for field effect transistor (FET) materials in printed electronics. However, there are currently two main problems, which are associated with inhomogeneity and poor thermal durability of these films. Here we report that liquid crystalline materials exhibiting a highly ordered liquid crystal phase of smectic E (SmE) can solve both these problems. We design a SmE liquid crystalline material, 2-decyl-7-phenyl-[1]benzothieno[3,2-b][1]benzothiophene (Ph-BTBT-10), for FETs and synthesize it. This material provides uniform and molecularly flat polycrystalline thin films reproducibly when SmE precursor thin films are crystallized, and also exhibits high durability of films up to 200 °C. In addition, the mobility of FETs is dramatically enhanced by about one order of magnitude (over 10 cm^2^ V^−1^ s^−1^) after thermal annealing at 120 °C in bottom-gate-bottom-contact FETs. We anticipate the use of SmE liquid crystals in solution-processed FETs may help overcome upcoming difficulties with novel technologies for printed electronics.

## Abstract (web summary)

Polycrystalline thin films of small molecules hold promise for organic thin-film transistors because of their large charge mobility, but are currently limited by poor film homogeneity and thermal durability. Here, Iino *et al*. design an ordered liquid crystal phase to overcome these two problems.

## Graphical abstract

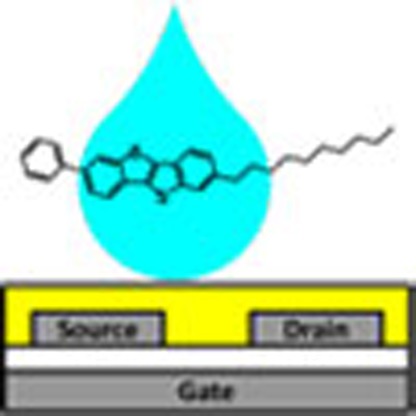

Recent research concerning materials for use in organic field effect transistors (OFETs), including small molecules and polymers, has been focused on solution-processed OFETs with applications in printed electronics. To allow for high mobility in these OFETs, significant effort has been directed towards the exploration of new, large π-conjugated molecules. Unfortunately, there is currently limited knowledge with regard to the design of molecular alignment in crystal lattices so as to achieve the desired level of mobility in crystalline thin films. As for solution-processability, OFET materials are often chemically modified with long hydrocarbon chains to improve their solubility in common organic solvents. In this manner, high mobilities (over 1 cm^2^ V^−1^ s^−1^) have been achieved in solution-processed OFETs fabricated with polycrystalline thin films of small molecules, including TIPS-pentacene^1^ and 2,7-dialkyl-[1]benzothieno[3,2-b][1]benzothiophene (dialkyl-BTBTs)^2^. Even higher mobilities of over 10 cm^2^ V^−1^ s^−1^ have been reported in strained or single-crystal thin films^3^,^4^,^5^. With regard to practical applications, however, the use of small molecules retains two deficits. The first is poor uniformity and rough surface morphology in solution-processed films resulting from recrystallization of OFET materials during solvent evaporation. This not only hinders reliable device performance and film fabrication but also results in wide variations in device performance. The other challenge is the poor thermal durability of the films, due to the low melting points brought about by chemical modification of the molecules with long hydrocarbon chains. These melting points are often in the vicinity of 100 °C and thus limit the thermal processes that are used for wiring and passivation during device fabrication. In practice, it is quite difficult to achieve both high solubility and good thermal durability simultaneously, since both are associated with the π–π interactions between OFET molecules. This represents a dilemma, due to the trade-off between the two factors.

Because of this problem, recent research has focused on π-conjugated main chain polymers^6^,^7^,^8^, which favor easy fabrication of uniform films and high thermal durability. These include a liquid crystalline polymer of poly(2,5-bis(3-alkylthiophene-2-yl)thieno[3,2-b]thiophenes) (PBTTT)^7^, donor–acceptor polymers of diketopyrrolopyrrole-based copolymers^9^, and the recently proposed polymer of poly[4-(4,4-dihexadecyl-4H-cyclopenta[1,2-b:5,4-b']dithiophen-2-yl)-alt-[1,2,5] thiadiazolo[3,4,-c]pyridine] (PCDTPT)^10^. These polymers do indeed show promise with respect to the formation of uniform films by wet processes and good thermal durability, but, to ensure high mobility, the resulting films must be thermally annealed at high temperatures in excess of 200 °C. In addition, there are often difficulties with regard to reproducible synthesis and purification of the polymers, control of molecular orientations towards both the substrate surface and the electrodes and crystallinity within the film.

To solve these problems and take full advantage of small molecules as an alternative to the polymers described above, we paid attention to the liquid crystal phase that often appears in OFET materials chemically modified with long alkyl chains^11^,^12^,^13^,^14^,^15^. In particular, our work focused on a highly ordered smectic E (SmE) mesophase in rod-like liquid crystals. Polycrystalline films of such OFET materials readily melt into droplets at their crystallization temperature when the OFET materials show less ordered liquid crystal phases, such as nematic, SmA^2^, or SmC phases. However, it is expected that such films will not transition to droplets at that temperature and thus the film shape will be retained when they show the SmE phase because of its solid-like nature. Furthermore, the SmE phase exhibits a herringbone structure in its molecular alignment. Since this structure is naturally maintained when the material is crystallized from the SmE phase, the resulting crystals exhibit two-dimensional conduction that favours high mobility^16^,^17^,^18^.

In this paper, we design and synthesize the SmE liquid crystalline material of 2-decyl-7-phenyl-benzothienobenzothiophene (Ph-BTBT-10, Fig. 1a) for OFETs and describe the phase transition behaviour, the crystallographic properties and the fabrication of its crystalline thin films by spin coating and their application to FET devices. All the results indicate high potential of Ph-BTBT-10 for FET applications, including solution processability for uniform polycrystalline thin films and high thermal durability of the films up to 200 °C. In addition, mobility of the FETs is dramatically enhanced by about one order of magnitude after thermal annealing at 120 °C and the highest mobility was 13.9 cm^2^ V^−1^ s^−1^ in bottom-gate and bottom-contact FETs. We conclude the use of SmE liquid crystals in solution-processed OFETs may help overcome upcoming difficulties with novel technologies for printed electronics.

## Result

### Molecular design of Ph-BTBT-10 and its liquid crystallinity

Based on our new strategy for the molecular design of highly ordered smectic liquid crystals (unpublished results), Ph-BTBT-10 was designed and synthesized: a large π-conjugated fused ring system of a benzothienobenzothiophene (BTBT) moiety was selected to achieve ready crystallization; in addition, a phenyl group was attached to the BTBT molecule to achieve molecular motion around the long molecular axis, thus providing ‘disorder' in the resulting molecular system required in the SmE phase; a long flexible hydrocarbon chain of the decyl group was also appended to the end of the BTBT moiety to facilitate formation of the liquid crystal phase. Furthermore, rather than the double side chains applied in the conventional molecular design of liquid crystals, we adopted a single side chain to generate possible asymmetric phase transition behaviour during the cooling and heating processes, such that we expected that the crystallization temperature during cooling would be far below the crystallization temperature associated with heating. As discussed in more detail further on, it is beneficial to the production of OFETs both to fabricate a precursor liquid crystalline film at lower temperatures and to maintain a crystalline film at higher temperatures.

**Figure 1:**
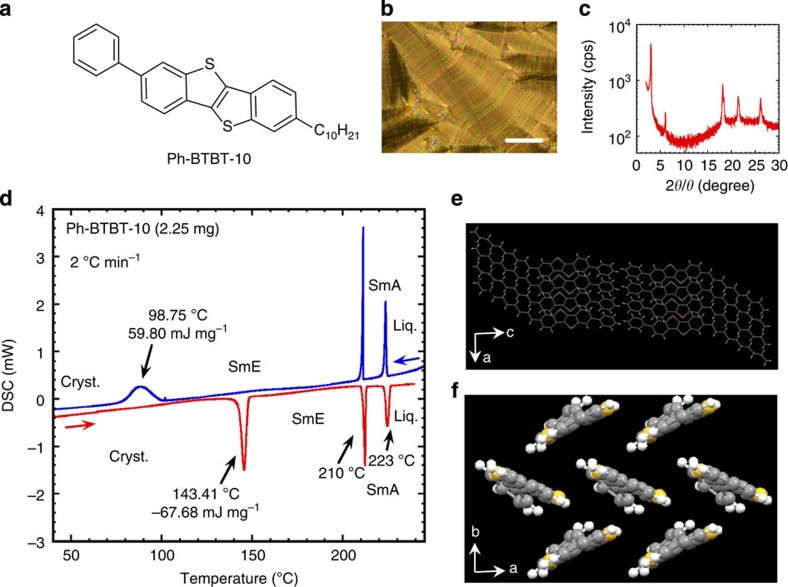
Basic characteristic of Ph-BTBT-10. (**a**) Chemical structure, (**b**) texture as determined by polarized optical microscopy at 150 °C, white bar indicates a scale of 50 μm in length, (**c**) XRD pattern of a liquid crystalline film exhibiting a polydomain texture acquired at 160 °C in an out-of-plain configuration, (**d**) DSC curves obtained at 2 °C min^−1^ and single-crystal structures of the (**e**) ac and (**f**) ab planes. Cryst., crystalline; Liq., liquid.

Ph-BTBT-10 exhibits two liquid crystal phases at low and high temperatures: the liquid crystalline phase at the low temperatures exhibited a typical fan-like texture with disclination lines as observed by polarized optical microscopy (Fig. 1b); furthermore, it had three peaks at ∼18°, 21° and 26° in the X-ray diffraction (XRD) pattern attributed to the rectangular structure, in addition to a peak at a small angle of ∼3° attributed to the smectic layer structure, as shown in Fig. 1c; on the other hand, the liquid crystalline phase at the high temperatures exhibited a fan-like texture without disclination lines as shown in Supplementary Fig. 1 and only a peak at a small angle of ∼3° in the XRD pattern. Thus, these were identified to be the SmE and SmA phases, respectively. As expected, Ph-BTBT-10 exhibited different phase transition behaviours, exhibiting the SmE phase at 99 °C during the cooling process and at 143 °C during the heating process, as shown in the differential scanning calorimetry (DSC) chart in Fig. 1d. This allowed us to fabricate a SmE film as a precursor to a crystalline film at temperatures far below 143 °C (that is, 100 °C or lower).

### Fabrication of polycrystalline thin films

**Figure 2:**
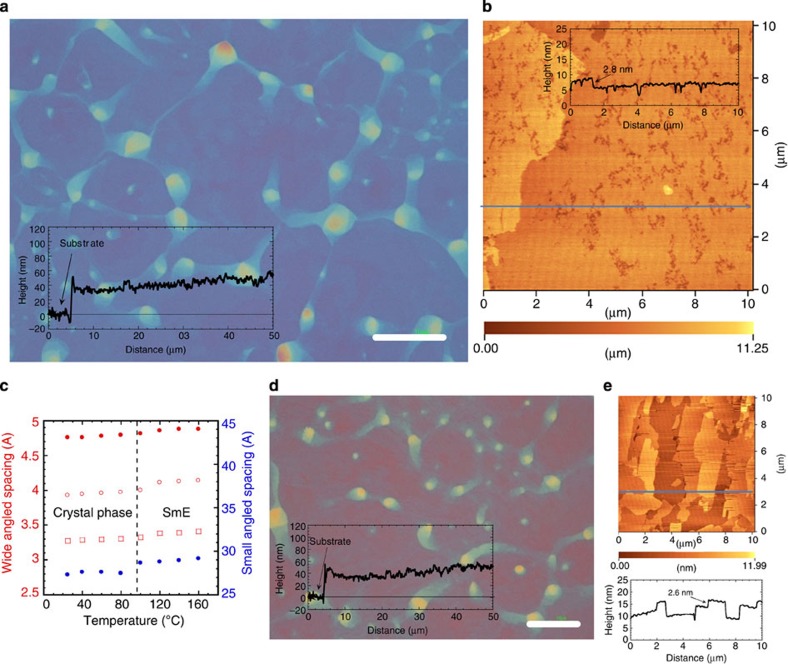
Characteristics of polycrystalline thin films of Ph-BTBT-10. The polycrystalline thin film were fabricated from a 0.5 wt% diethylbenzene solution at ∼110 °C, (**a**,**d**) texture as determined by optical microscopy and (**b**,**e**) AFM images. Insets show cross-sectional profiles observed by (**a**,**d**) confocal laser scanning microcopy and (**b**,**e**) AFM. (**a**,**b**) As-coated polycrystalline film and (**d**,**e**) after thermal stress at 150 °C for 5 min. (**a**,**d**) White bars indicate scale of 20 μm in length. (**c**) Four d-spacings of Ph-BTBT-10 in crystal and SmE phase in wide- and small-angle regions as a function of temperature.

Ph-BTBT-10 polycrystalline thin films were fabricated by spin coating a diethylbenzene solution at the SmE phase temperature of 110 °C and cooling the SmE films to room temperature. The resulting polycrystalline films were very uniform, which are very similar to those fabricated from less-ordered liquid crystalline films previously reported^19^,^20^, but contained no cracks and maintained the crystal state up to 143 °C. The different colors in the film shown in Fig. 2a are due to interference in the samples consisting of a Ph-BTBT-10 layer of about 50 nm and silicon dioxide of 300 nm on Si-substrate, indicating that the film has a small variation in thickness within a film. The absence of cracks in the polycrystalline thin films is attributed to similar thermal expansion coefficients of lattices within the molecular layer in the SmE and crystal films, which was confirmed by continuous decrease of three d-spacings corresponding to SmE structure as a function of temperature structure, in spite of the phase transition from SmE to crystal phases, as shown in Fig. 2c and Supplementary Fig. 2. These films are therefore very different from the cracked polycrystalline thin films previously fabricated from SmA films of a dialkyl-BTBT derivative^20^. Atomic force microscope (AFM) image of the polycrystalline thin film shows wide terrace structures with steps of ∼2.8 nm, as shown in Fig. 2b, corresponding to the molecular length along the long molecular axis of Ph-BTBT-10 as shown in Supplementary Fig. 3. This indicates that the Ph-BTBT-10 molecules are aligned perpendicular to the substrate. Thanks to the solid-like SmE phase similar to crystal phase, the resulting films not only maintained the film shape but also molecular flatness even after heating at 150 °C for 5 min, as shown in Fig. 2d,e.

### Top contact FETs fabricated with Ph-BTBT-10

**Figure 3:**
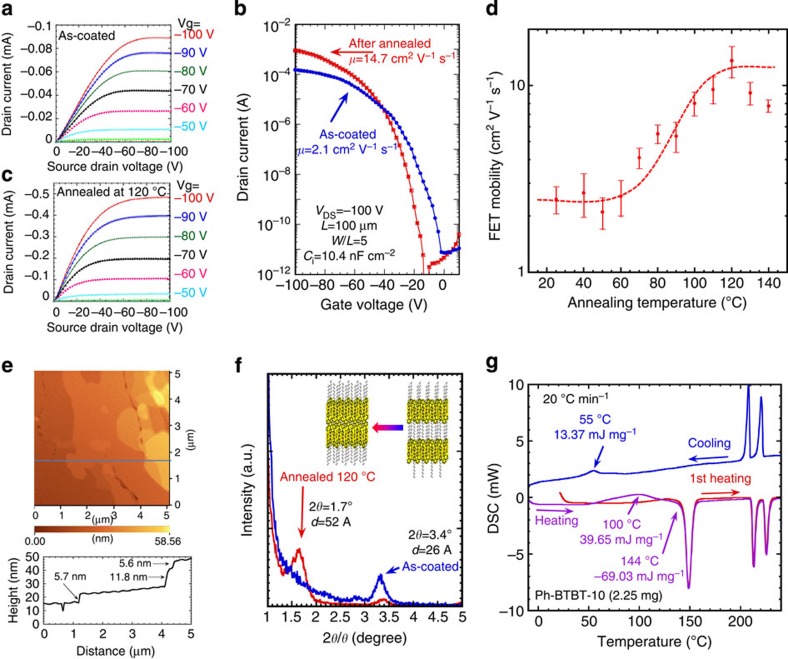
Characteristics of top-contact FET and its change after thermal annealing. Characteristics of top-contact type FETs made using polycrystalline thin films of Ph-BTBT-10 fabricated from 0.5 wt% *p*-xylene solution in the SmE phase at ca., 80 °C. Output characteristics of FETs fabricated using the polycrystalline thin films (**a**) as-coated and (**c**) after thermal annealing at 120 °C for 5 min, (**b**) transfer characteristics of FETs fabricated with polycrystalline thin films both as-coated and after thermal annealing, (**d**) FET mobility as a function of annealing temperature, the error bars calculated from the standard deviations over 10 samples in each annealing temperature, (**e**) a topographic image of the polycrystalline thin films after annealing at 120 °C, as obtained by AFM, (**f**) out-of-plane small-angle XRD patterns for crystalline films as-coated and after annealing, with a schematic illustration of the crystalline structure and (**g**) DSC data for Ph-BTBT-10 during fast cooling and heating at 20 °C min^−1^.

Bottom-gate and top-contact type FETs were fabricated with polycrystalline thin films of Ph-BTBT-10 on SiO_2_ (300 nm)/Si-substrates to allow the characterization of this compound as an OFET material. These FETs exhibited good p-channel performance, as summarized in Fig. 3a. Figure 3b demonstrates that the FET mobility under ambient condition was 2.1 cm^2^ V^−1^ s^−1^. Surprisingly, the FET transfer characteristics were significantly improved after brief thermal annealing at 120 °C for 5 min. Following this annealing, the drain current dramatically increased to 10^−3^ A and the FET mobility in the saturation region was also enhanced, reaching a value of 14.7 cm^2^ V^−1^ s^−1^, as shown in Fig. 3b,d.

### Transition from monolayer to bilayer structure

**Figure 4:**
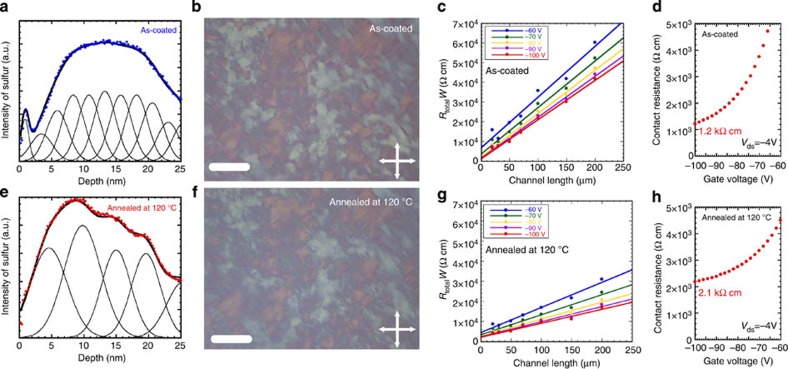
Characteristics of the polycrystalline thin films and contact resistance. Depth profile of sulfur atom (**a**) before, (**e**) after thermal annealing at 120 °C observed by TOF-SIMS. Depth axis was determined by sputtering rate. Polarized optical microscope images of polycrystalline thin films of Ph-BTBT-10 (**b**) before and (**f**) after thermal annealing at 120 °C for 5 min. White bars indicate scale of 20 μm in length. Contact resistance of FETs fabricated with polycrystalline thin film of Ph-BTBT-10 (**c**,**d**) before and (**g**,**h**) after thermal annealing at 120 °C for 5 min evaluated by transfer line method.

The AFM observations revealed that thermal annealing promotes a change in the step structure from monolayer steps of 2.8 nm to bilayer steps of 5.7 nm, as can be seen in the surface profile presented in Figs 2b and 3e. Furthermore, the XRD patterns exhibit a distinct change in the peaks at 3.4° and 1.7° before and after thermal annealing, as shown in Fig. 3f. These variations suggest that the monolayer structure with a thickness of 26 Å before thermal annealing was transformed into a bilayer structure with a thickness of 52 Å, as shown in the schematic illustration in Fig. 3f. Figure 3d summarizes the improvements in FET mobility as a function of annealing temperature, from which it is evident that enhancements start at ∼70 °C and saturate in the vicinity of 120 °C. This variation coincides with an observed exotherm in the temperature range from 70 to 120 °C before a large endotherm associated with the transition from the crystal to the SmE phase at 144 °C during the heating process, as shown in Fig. 3g. These results demonstrate that the structural changes occurring in the crystal during thermal annealing may be attributed to a crystal-to-crystal phase transition that generates the same crystal structure in Ph-BTBT-10 recrystallized from solutions. In fact, a crystallographic study of a Ph-BTBT-10 single crystal formed from a toluene solution revealed that each unit cell contains two molecules oriented head to head at the inter layer, and that the Ph-BTBT moiety is aligned in a herringbone structure within a molecular layer, as shown in Fig. 1e,f. Thus, it was concluded that the observed significant improvements in the FET mobility result from the structural change of the Ph-BTBT-10 from a monolayer to a bilayer structure. This phenomenon was confirmed by the compositional depth profile of thin films obtained from time-of-flight secondary ion mass spectrometric (TOF-SIMS) studies before and after thermal annealing as shown in Fig. 4a,d, respectively, in which the depth profiles of sulfur are well fitted with 2.5 and 5 nm thick sulfur-containing layers Gaussian-distributed, respectively. On the other hand, the bilayered polycrystalline thin films were retransformed when heated over 143 °C, which is the phase transition temperature from crystal to SmE phases as shown in Supplementary Fig. 4. This conclusion is also reinforced by the observation that neither a change in the 10 μm grain size nor a reduction in the contact resistance of ∼1 kΩ cm at the interface with the source/drain electrodes in FETs was observed after thermal annealing as shown in Fig. 4b,e and c,f, respectively. With regard to the design of materials for OFETs, a bilayer structure consisting of mono-alkylated π-conjugated molecules, representing a quasi-extended large π-conjugated system, evidently offers a new strategy for achieving high mobility without sacrificing solubility. This approach is different from the conventional strategy of extending the π-conjugated system in existing molecules.

### Bottom contact FETs fabricated with Ph-BTBT-10

**Figure 5:**
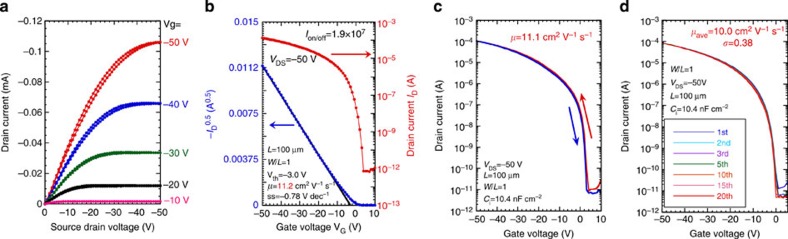
Characteristics of bottom-contact FETs after thermal annealing. Characteristics of bottom-gate, bottom-contact type FETs made with polycrystalline thin films of Ph-BTBT-10 after thermal annealing at 120 °C. (**a**) output characteristics and (**b**–**d**) transfer characteristics. Transfer characteristics focused on (**c**) hysteresis and (**d**) repeated measurement properties. dec, decade.

Based on the apparent promise of Ph-BTBT-10 as a solution-processed OFET material, we fabricated OFETs on a SiO_2_ (300 nm)/Si substrate in the more practical device configuration of bottom-gate and bottom-contact type OFETs, and evaluated the device performance and reliability obtained from the present film fabrication process. To ensure good electrical contact with Ph-BTBT-10 in the bottom-contact configuration, we modified the Au electrodes used for the source and drain with pentafluorobenzenethiol (PFBT) and obtained a small contact resistance of 156 Ω cm, which is one order of magnitude smaller than that of the top-contact type OFETs as shown in Supplementary Fig. 5. Figure 5a,b present the typical output and transfer characteristics of bottom-gate and bottom-contact OFETs, respectively. The mobility obtained after thermal annealing was estimated to be 11.2 cm^2^ V^−1^ s^−1^ in the saturated region, while *V*_th_ was −3 V and the subthreshold swing was 0.78 V per decade.

### Reproducibility and reliability of FET devices

**Figure 6:**
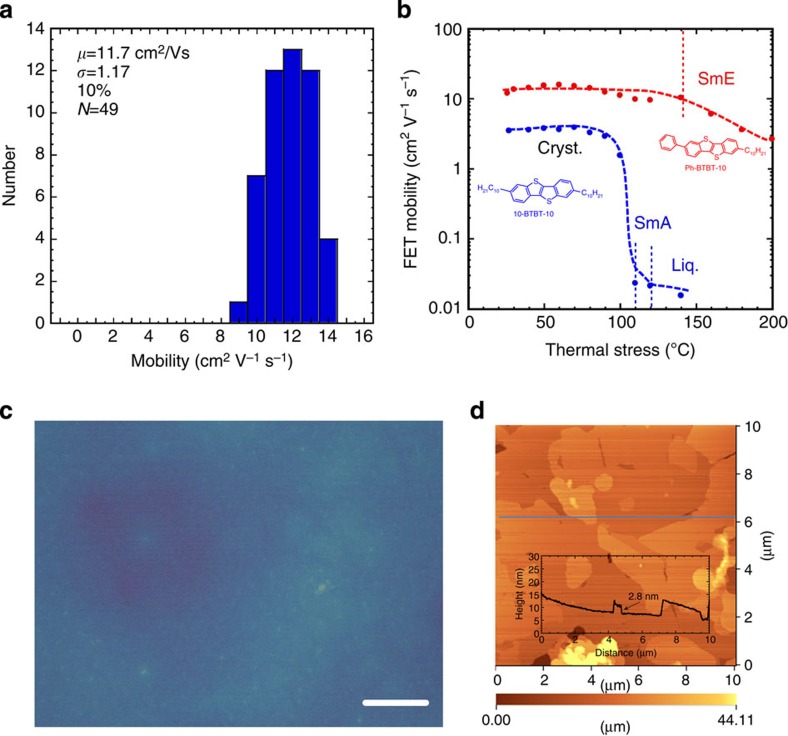
Reliability of FETs and fabrication of uniform thin films at 40 °C. FET characteristics of bottom-gate, bottom-contact type FETs made with polycrystalline thin films of Ph-BTBT-10 after thermal annealing at 120 °C. (**a**) Variations of FET mobility in five films. Thermal durability properties of FET fabricated with polycrystalline thin films of Ph-BTBT-10. (**b**) FET mobility as a function of stress temperature. Polycrystalline thin films of Ph-BTBT-10 fabricated from a *p*-xylene solvent mixture. (**c**) Optical microcopy texture and (**d**) AFM image. (**c**) White bar indicates a scale of 20 μm in length. Cryst., crystalline; Liq, liquid.

To evaluate reliability of FET performance, we investigated hysteresis of FET performance during forward and reverse operation as well as repeated measurements of the characteristics. FET characteristics are hardly changed during forward and reverse operation as a function of gate voltage and repeated measurement as shown in Fig. 5c,d, respectively. Furthermore, to evaluate reproducibility of FET performance in both device to device and sample to sample a total of 49 bottom-gate and bottom-contact transistors on five substrates were fabricated and the transfer and output characteristics of each transistor were measured. As shown in Fig. 6a, the transistors exhibit minimal variations in FET mobility, with an average mobility of 11.7±1.2 cm^2^ V^−1^ s^−1^ (9.5–13.9 cm^2^ V^−1^ s^−1^) for the 49 devices. Table 1 summarizes the variations in performance of FETs fabricated with five films, and shows a film-to-film variation of ∼10%. We attribute this variation to minor differences in the uniformity and surface smoothness of the films resulting from the fabrication process. It should be noted that, even though the present study employed polycrystalline OFETs, the high mobilities observed here (above 10 cm^2^ V^−1^ s^−1^) are comparable to the values normally obtained from single-crystal OFETs^3^,^21^,^22^,^23^,^24^. Considering that it could potentially be necessary to fabricate millions of FETs for display applications, polycrystalline OFETs may have an advantage over single-crystal OFETs. This advantage arises because variations in FET performance in single-crystal OFETs is determined by the quality of each single-crystal film, which in turn may be greatly affected by the formation of twins and/or multiple grains, as well as the crystal orientation towards the source and drain electrodes.

The thermal durability of the present FETs was also evaluated. Figure 6b summarizes FET mobility values as measured under ambient conditions after thermal stress at a given temperature for 5 min, as a function of the stress temperature. The FET mobility values exhibit little change and remain above 10 cm^2^ V^−1^ s^−1^ up to 140 °C, and a value of 2.6 cm^2^ V^−1^ s^−1^ is obtained even after thermal stress is applied at 200 °C for 5 min. In contrast, the mobility in FETs fabricated with polycrystalline thin films of 2,7-didecylbenzothienobenzothiophene (10-BTBT-10) abruptly decreases after thermal stress is applied above 100 °C, at which temperature the crystal film was observed to melt into droplets due to the phase transition to a liquid-like, less-ordered phase of SmA and the FETs ceased to function^25^,^26^.

As described above, fabrication of crystalline films from precursor SmE films is considered to be the key factor accounting for the minor variations in FET performance found in this work. Surprisingly, uniform crystalline thin films could be fabricated at temperatures down to 40 °C below the temperature associated with the SmE phase, as shown in Fig. 6c,d, when employing spin coating from a *p*-xylene:chloroform solution (4:1 v/v) of Ph-BTBT-10. The exact reason for this phenomenon is not yet known, but it is possible that the self-organization of liquid crystalline Ph-BTBT-10 molecules may contribute to the formation of a uniform film at that temperature, in spite of the competing recrystallization process during solvent evaporation.

In conclusion, we successfully fabricate OFETs using polycrystalline thin films obtained from Ph-BTBT-10 as a SmE liquid crystal. This material is evaluated with regard to its potential for practical applications in solution-processed OFETs. We demonstrate that the unique nature of the SmE phase not only enable us to fabricate uniform and molecularly flat films, giving only minor variations in FET performance between devices and between samples, but also improves the thermal durability of the films up to 200 °C, such that small-molecule OFET materials may offer the same advantages as polymeric OFET materials. The new molecular design strategy adopted with Ph-BTBT-10 is applicable to different types of extended *π*-conjugated system and should allow tailoring of transistor materials for particular printing technologies. The discovery of a dramatic enhancement of FET mobility up to 13.9 cm^2^ V^−1^ s^−1^, resulting from the phase transition from a monolayer to a bilayer crystal structure in mono-alkylated liquid crystalline molecules may lead to the possibility of designing new materials for the burgeoning field of printed electronics.

## Methods

### Materials

Ph-BTBT-10 was synthesized by the Suzuki coupling reaction between 2-decyl-7-iodo-[1]benzothieno[3,2-b][1]benzothiophene(I-BTBT-10) and phenyl borate as shown in Supplementary Fig. 6. The phase transition behaviour of this material was characterized by DSC (DSC-60, Shimadzu), polarized optical microscopy (OPTIPHOT2-POL, Nikon and FP82HT hot stage, Mettler Toledo) and XRD (RAD-2X, Rigaku). Single crystals of Ph-BTBT-10 were prepared by recrystallization from toluene and analyzed by XRD (R-AXIS RAPID II/R, Rigaku) using CrystalStructure 4.0 software (Rigaku) to determine the structure.

### Fabrication of thin films

Polycrystalline thin films of Ph-BTBT-10 were fabricated by spin coating at a given temperature, for which we preheated the solution, a glass pipet and a substrate on a thick glass substrate of 1.1 mm for a big heat capacity in order to make sure the spin coating at that temperature before pipetting a small amount of the solution onto the substrate and turning on the spin coater. We carried out this procedure as quickly as we could in ∼3 s, while the substrate temperature was monitored by an infrared thermometer calibrated in advance. In the spin coating at high temperature range of over ∼100 °C, spin coating was carried out either in a vacuum oven or under illumination with an infrared lamp. The temperatures of spin coating described in this paper indicate the initial temperature of substrate before spin coating at 3,000 r.p.m. The spin-coating solution of Ph-BTBT-10 was 1,4-diethylbenzene or *p*-xylene and its concentration was ∼0.5 or 0.75 wt%. The resulting films were evaluated by optical microscopy, confocal laser scanning microscopy (VK-9700, Keyence), and atomic force microscopy (AFM, SPA300 DFM in tapping mode, Seiko).

### Fabrication and evaluation of FET

To fabricate a bottom-gate top-contact type FET, gold electrodes were deposited on the polycrystalline thin films on SiO_2_ (∼300 nm)/p^+^-Si substrates. The channel length (*L*)/width (*W*) were 100/500 μm or 100/100 μm in normal measurements, 200/1,000 μm, 150/750 μm, 100/500 μm, 70/350 μm, 50/250 μm, 30/150 μm and 20/100 μm in transfer line method. In bottom-gate bottom-contact type FET, polycrystalline thin film was fabricated by spin coating on the substrate with source-drain electrodes deposited by vacuum evaporator in 3 × 10^−4^ Pa on SiO_2_ (∼300 nm)/p^+^-Si substrates. The gold source and drain electrodes were modified with PFBT in a glove box. Gold electrodes were soaked in 0.1 mol l^−1^ PFBT with anhydride ethanol for 20 min and then clean the substrate by ethanol and acetone.

The FET performance was characterized by two source measurement units (8,252, ADCMT). The mobility (*μ*) was calculated by plotting the square root of the source-drain current (*I*_ds_) versus gate voltage (*V*_g_) and using the equation from the saturated region,


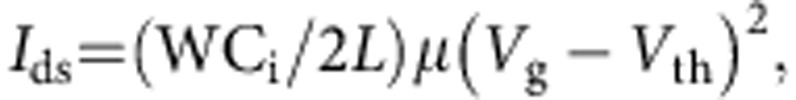


where C_i_ is capacitance of gate insulator and *V*_th_ is threshold voltage. One substrate, ∼1.5 × 1.5 mm, has 10 FET devices. *σ* is calculated s.d. in the devices.

### Evaluation of thin films by TOS-SIMS

The compositional depth profile of sulfur atoms in the polycrystalline thin film was studied by TOF-SIMS (ION-TOF GmbH, TOF-SIMS 5-100-AD); the sample surface was probed by 60 keV Bi_3_^++^ cluster ions and sputtered by Cs^+^ ions at an energy of 2 keV; the mass spectra were measured in the negative secondary ion mode. Depth axis was determined by sputtering rate.

## Author contributions

H.I. fabricated thin films, determined the crystal structures, phase transition behaviors, and FET. characteristics, collected all data and co-wrote this manuscript. T.U. synthesized and purified Ph-BTBT-10. J.H. directed the overall research project, designed the molecules and co-wrote this manuscript. Each of the authors discussed the results and commented on the manuscript.

## Additional information

**How to cite this article:** Iino, H. *et al*. Liquid crystals for organic thin-film transistors. *Nat. Commun*. 6:6828 doi: 10.1038/ncomms7828 (2015).

## Supplementary Material

Supplementary Information Supplementary Figures 1-6, Supplementary Notes 1-5, Supplementary Methods and Supplementary References

## Figures and Tables

**Table 1:** Variations of FET mobility in five films.

Sample No.	*μ* (cm^2^ V^−1^ s^−1^)	*σ*	*σ*/*μ* (%)
A	11.0	0.8	7
B	12.1	1.0	8
C	12.0	1.1	10
D	11.3	1.3	11
E	12.5	1.0	8

*μ*, *σ* and *σ*/*μ* indicate the average mobility, s.d. and ratios of s.d., respectively.
