# Supplementary material for: Liquid crystals for organic thin-film transistors
Source: Nat Commun. 2015 Apr 10;6:6828. doi: 10.1038/ncomms7828 (PMC4403349; doi:10.1038/ncomms7828)
Supplement: Supplementary Information — Supplementary Figures 1-6, Supplementary Notes 1-5, Supplementary Methods and Supplementary References [file ncomms7828-s1.pdf]

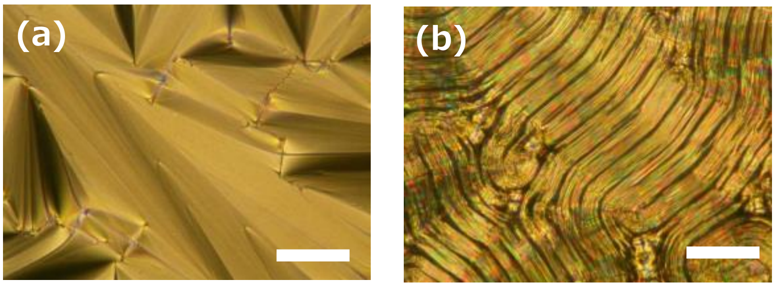

Supplementary Figure 1 | Polarized optical microscope textures of Ph-BTBT-10 in a liquid crystal cell.

(a) SmA and (b) crystal phases at 220 and 26°C, respectively. White bars indicate a scale of 50μm in length.

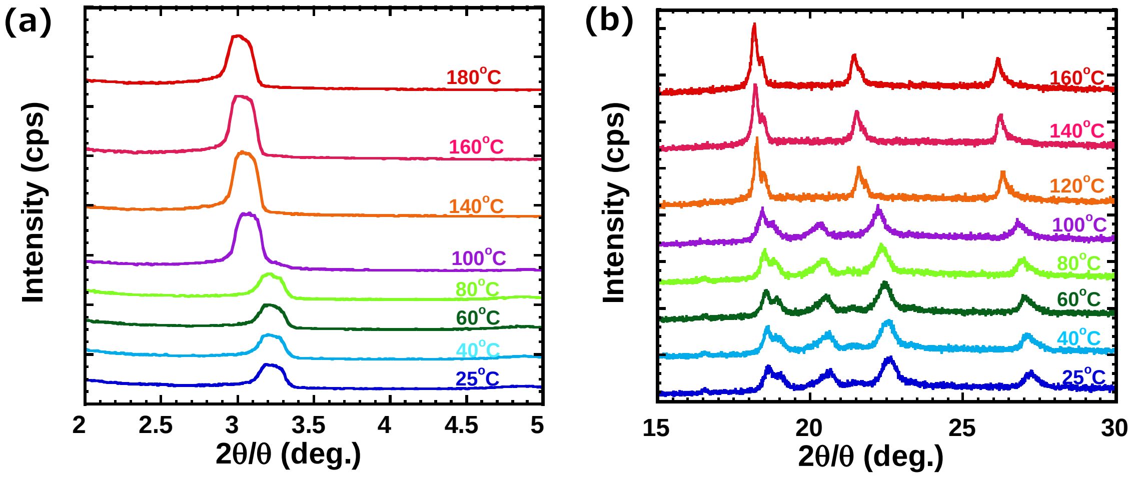

Supplementary Figure 2 | XRD patterns of Ph-BTBT-10 at several temperatures.

In (a) small angel and (b) wide angel regions.

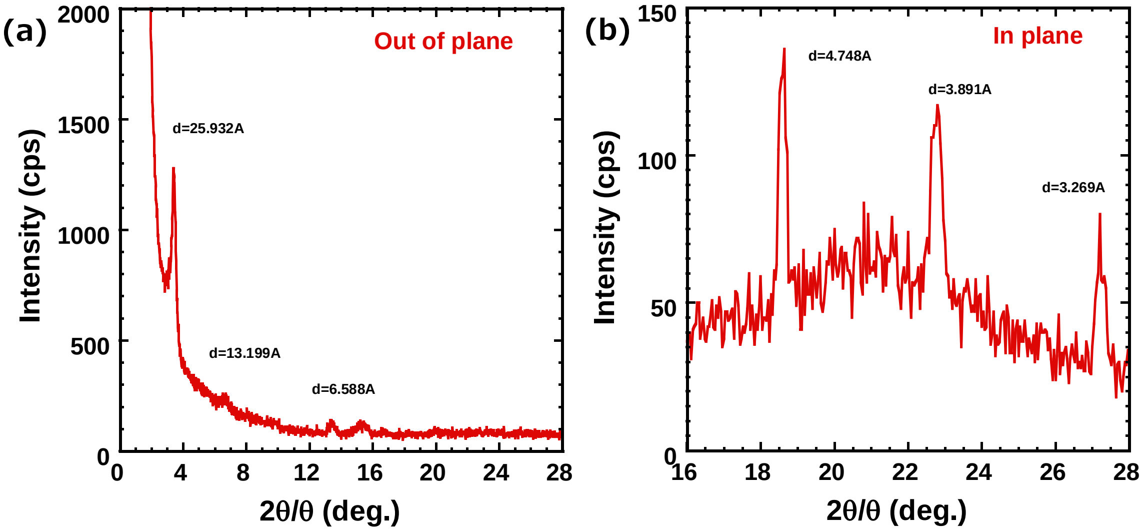

Supplementary Figure 3 | XRD patterns of polycrystalline thin film of Ph-BTBT-10.

The polycrystalline thin films were fabricated in SmE phase at ca. 80 ^{o}C. XRD patterns were measured in (a) out-of-plane and (b) in-plane.

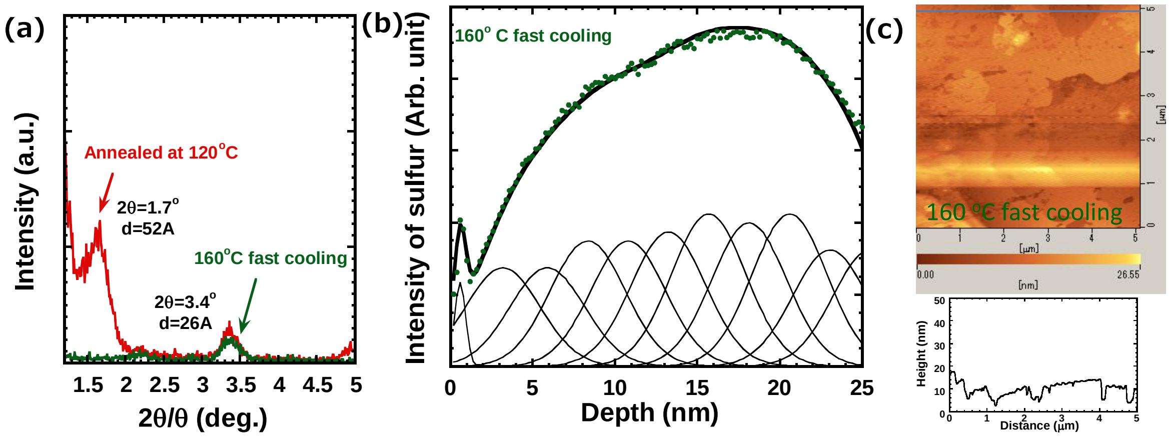

Supplementary Figure 4 | Molecular alignment of Ph-BTBT-10 after heating at SmE phase.

The polycrystalline thin film of Ph-BTBT-10 after heating at 160 ^{o}C in SmE phase. (a) The XRD pattern in small angle, (b) depth profile of sulfur atom observed by TOF-SIMS, and (c) surface profile observed by AFM.

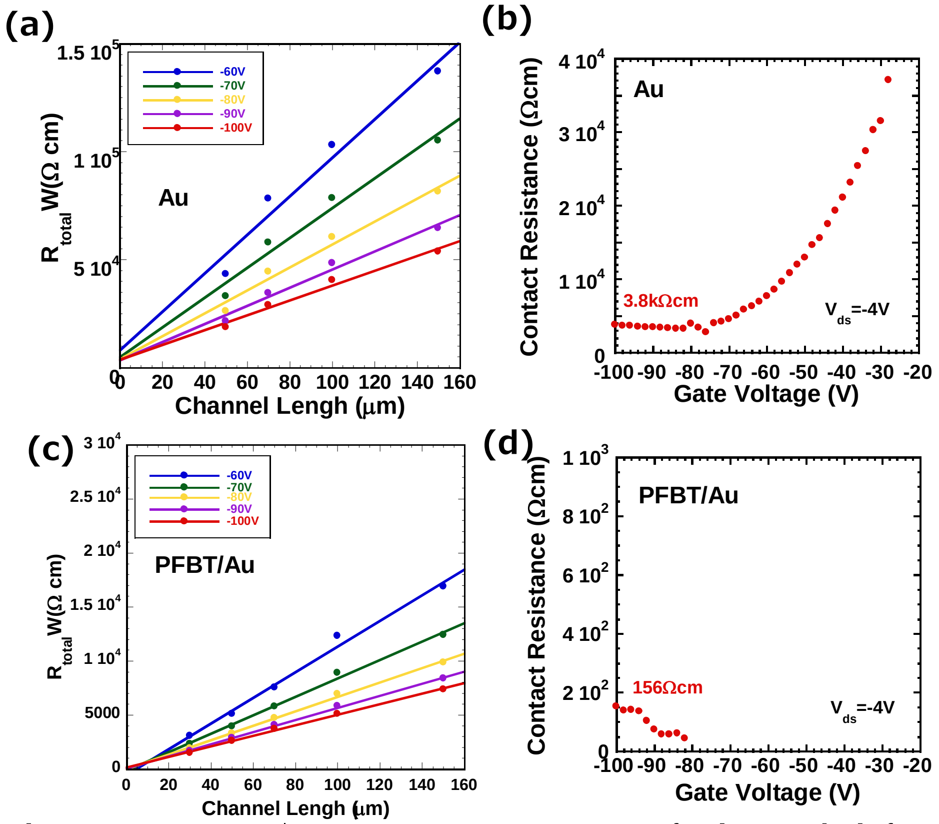

Supplementary Figure 5 | Contact resistance in a transfer line method for a bottom-gate bottom-contact FET.

(a,b) bare gold electrode and (c,d) PFBT-modified Au electrodes.

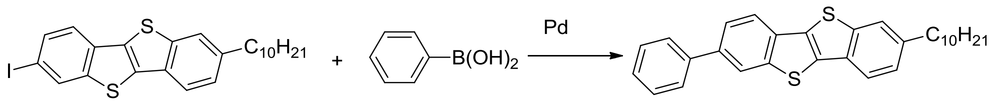

Supplementary Figure 6 | Synthesis of 2-decyl-7-phenyl-[1]benzothieno[3,2-b][1]benzothiphene.

Supplementary Note 1 | Phase transtion behavior of Ph-BTBT-10

The phase transition temperatures were determined by differential scanning calorimetry (DSC: Shimadzu 60). Their liquid crystal phases were identified by the textures observation under a polarized optical microscope (Nikon OPTIPHOT2-POL and Mettler Toledo FP82HT Hot stage).

Ph-BTBT-10, exhibited two mesophases from 143 to 223 ^{o}C as shown in the DSC chart of Fig. 1(d). The polarized optical microcopy textures at 150, 220 and 26 ^{o}C showed disclination line, fan like patterns, and strip texture with cracks as shown in Fig. 1(b), Supplementary Fig. 1(a), and Supplementary Fig. 1(b), respectively. These textures strongly indicate SmE, SmA, and crystal phases, respectively. Molecular length of Ph-BTBT-10 is 27.0Å, which is calculated by geometrical optimization with MM2.

Supplementary Note 2 | Structure transition of Ph-BTBT-10 from SmE to crystal phases

The liquid crystal and crystal structures of Ph-BTBT-10 are evaluated by X-ray diffraction (XRD) analysis using Rigaku RAD-2X diffractometer with CuKα radiation. The rectangular molecular alignment in SmE phase was very similar to that in crystal phase, and the d-spacing of the molecules within a layer in the wide angles continuously was changed without its abrupt shrinkage in the phase transition from the SmE to crystal phases, while the d-spacing between layers was shortened as shown in Fig. 2(c) and Supplementary Fig. 2. This result explained why no crake was formed in the polycrystalline thin films of Ph-BTBT-10.

Supplementary Note 3 | Molecular alignment of polycrystalline thin films

The molecular alignments of the polycrystalline thin films on a SiO_2/Si-substrate were evaluated by X-ray diffraction (XRD) analysis, using Rigaku RAD-2X diffractometer with CuKα radiation for out-of-plane measurement and Bruker AXS Co. Ltd. D8 Discover μHR for in-plane measurements.

XRD spectra of out-of-plane and in-plane of the polycrystalline thin films fabricated at 82 ^{o}C indicated that molecules are aligned perpendicular on the substrate as shown in Supplementary Fig. 3.

Supplementary Note 4 | Crystal structure of polycrystalline thin film after heating to SmE phase

In order to clarify the mono-layer or bi-layer structures in a bi-layered film of Ph-BTBT-10 after heating at the temperature of over 143 ^{o}C, which is the phase transition temperature from crystal to SmE phases of Ph-BTBT-10 in heating process, we evaluated the thin films of Ph-BTBT-10 after thermal heating at 160 ^{o}C and fast cooling to room temperature by XRD measurement in small angle region, TOF-SIMS, and AFM images. XRD result showed a peak corresponding to mono-layer structure as shown in Supplementary Fig. 4(a). Furthermore, TOF-SIMS result showed no structure in the depth profile of sulfur atoms as shown in Supplementary Fig. 4(b). This indicates the film had the mono-layer structure as in the case of spin-coated films at a temperature of SmE phase. In fact, the films showed 2.5 nm steps for a mono-layer structure as shown in Supplementary Fig. 4(c) of the surface profile observed by AFM.

Supplementary Note 5 | Contact resistance bottom-gate bottom-contact FET

The HOMO level of Ph-BTBT-10 was estimated to be 5.6eV by cyclic voltammetry which is the same as that of dialkyl-BTBT derivatives. In even bottom-contact FET, the contact resistance with gold electrodes was small, i.e., 3.8kΩcm as shown in Supplementary Fig. 5(a, b), which is much smaller than the contact resistance of dialkyl-BTBT derivatives reported to be 10-100 kΩcm.[1] However, Ph-BTBT-10 FET has high mobility of over 10 cm^2/Vs and high drain current, 10^{-3}A (V_{DS}=-100V, W=500μm) as shown in Fig. 3(b), and the total resistance of 5kΩcm, which was comparable to the contact resistance of several kΩcm. Because of these, FET showed non-ideal characteristics. In order to improve the FET performance, we adopted the bottom-gate bottom-contact FET with Au electrode treated with pentafluorobenzenethiol (PFBT) in order to improve the contact resistance. PFBT-treated Au electrodes gave a considerably small contact resistance of ca., 150 Ωcm as shown in Supplementary Fig. 5(c, d), while bare Au electrodes gave a contact resistance of 3.8kΩcm, leading to improved FET performance as shown in Fig. 5.

Supplementary Methods

1. Synthesis of 2-decyl-7-phenyl-[1]benzothieno[3,2-b][1]benzothiphene

2-decyl-7-phenyl-[1]benzothieno[3,2-b][1]benzothiphene was synthesized by the coupling reaction of 2-decyl-7-iodo-BTBT with either the corresponding boronic acid or triphenylstannane in the presence of palladium catalyst as described below in detail as shown in Supplemetary Scheme 1. 2-decyl-7-iodo-BTBT was prepared by the modified Sandmeier reaction of 7-decyl-BTBT-2-amine,[2] which was prepared by nitration of 2-decyl-BTBT and successive reduction with tin power and hydrochloric acid.

2-Decyl-7-phenyl-[1]benzothieno[3,2-b][1]benzothiophene (Ph-BTBT-10) (I)

2M potassium phosphate aqueous solution (0.45ml) and phenyl boronic acid (Tokyokasei, 110mg, 0.9 mmol) was added to 8ml dioxane solution containing 2-decyl-7-iodo-BTBT (228mg, 0.45mmol), and then the resulting solution was bubbled with argon for 20 min. And then, terakis(triphenylphosphine) palladium (Tokyokasei, 30mg, 0.025mmol) and trictclohexylephosphine (Wako Pure Chemicals, 13mg, 0.045mmol) were added to the solution, and then the mixture was heated at 95°C for 22hours while stirring. The resulting reaction mixture was cooled, diluted with chloroform, and washed with sodium chloride solution (10ml). The organic layer was separated and dried on anhydrous magnesium sulfate overnight. Then, the solvent was evaporated and the resulting solid (293mg) was recrystallized from toluene to give 2-decyl-7-phneyl-[1]benzothieno[3,2-b][1]benzothiophene (130mg) as slightly brown colored crystal in 63% yield.

Nuclear magnetic resonance (NMR) spectra were obtained in deuterated chloroform with a JEOL Lambda 500 operating at 500MHz for ^{1}H and a Bruker biospin AVANCE III 400 operating at 100 MHz for ^{13}C with TMS as internal reference; chemical shifts (δ) are reported in parts per million. High resolution mass spectrometry (HRMS) were obtained with double-focusing magnetic sector mass spectrometer JEOL JMS-700.

^{1}H NMR (500MHz, $CDCl_3$): δ 8.12(d, 1H, J 1.8Hz, H-6), 7.92(d, 1H, J 8.2Hz, H-9), 7.79(d, 1H, J 7.8Hz, H-4), 7.73(br. s, 1H, H-1), 7.69(d x 2, 3H, H-8, 2', 6' ('denote Ph)), 7.49(t, 2H, J ~8Hz, H-3', 5'), 7.38(tt, 1H, J >1, ~8Hz, H-4'), 7.29(dd, 1H, J >1, 7.8Hz, H-3), 2.77(t, 2H, J ~7Hz, $ArCH_2$), 1.70(quint. 2H, J ~7Hz, $ArCH_2CH_2$), ~1.2 ~ 1.4(m, 14H, CH_2 x 7), 0.88(t, 3H, J ~7Hz, CH_3). ^{13}C NMR (100MHz, $CDCl_3$): δ 142.97, 142.75, 140.88, 140.50, 138.18, 133.77, 132.44(×2), 131.14, 128.93(×2), 127.44, 127.36(×2), 126.00, 124.48, 123.40, 122.36, 121.59, 121.28, 36.18, 36.93, 31.68, 29.64, 29.62, 29.54, 29.34(×2), 22.70, 14.09. HRMS(m/z): $[M]^+$ calcd. For $C_{30}H_{32}S_2$: 456.1945; found: 456.1955.

Supplementary References

[1] Kano, M., Minari, T., Tsukagoshi, K., Improvement of subthreshold current transport by contact interface modification in p-type organic field-effect transistors. *Appl. Phys. Lett.*, **94**, 143304 (2009).

[2] Kosata, B., Kozmik, V., Svoboda, J., Reactivity of [1]benzothieno[3,2-b][1]benzothiophene -electrophilic and metallation reactions, *Collect. Czech. Chem. Commun.* **67**, 645-664 (2002).
